# DEPLOYING TELEWELLNESS PROGRAMS IN THE COMMUNITY: BARRIERS, FACILITATORS, AND POTENTIAL

**DOI:** 10.1093/geroni/igae098.1542

**Published:** 2024-12-31

**Authors:** Tracy Mitzner, Elena Remillard, Kara Mumma

**Affiliations:** Person in Design, Atlanta, Georgia, United States; Georgia Institute of Technology, Atlanta, Georgia, United States; Georgia Institute of Technology, Atlanta, Georgia, United States

## Abstract

‘Telewellness’ programs use technology (e.g., video conferencing software) to facilitate remote participation in exercise classes. These programs hold great potential to expand access to physical and mental health benefits of group exercise classes for older adults, especially those with mobility disabilities. We translated the Tai Chi for Health Institute’s in-person, evidence-based tai chi program, Tai Chi for Arthritis, to be a virtual experience (via Zoom) that is both socially engaging and inclusive of people with a mobility disabilities. The ‘Tele Tai Chi’ clinical trial (NCT04696887) explored to what extent this program may increase social interaction and positive health behaviors among older adults with long-term mobility disabilities; data analysis is in progress. The next phase of this project is focused on delivering our Tele Tai Chi program in real world, community-based settings (e.g., senior centers, senior living communities). This session will present findings from interviews with subject matter experts (e.g., senior center coordinators, administrators from Area Agencies on Aging, tai chi instructors) on perceived barriers to delivering Telewellness classes (e.g., safety, staffing, demands, technical support), as well as potential facilitators to address them. We will also discuss the need for different models of Telewellness (e.g., hybrid classes with attendees both in-person and online) that align with the community’s unique characteristics, preferences, and support needs.

